# An explainable graph retrieval augmented generation framework for personalized nutrition recommendation

**DOI:** 10.3389/frai.2026.1808444

**Published:** 2026-04-30

**Authors:** Varrsan Dindukurthi, Dhruv Jain, Anubhava Tripathi, Jagan Mohan Obbineni, Ilanthenral Kandasamy

**Affiliations:** 1 School of Computer Science and Engineering (SCOPE), Vellore Institute of Technology, Vellore, Tamil Nadu, India; 2 VIT School of Agricultural Innovations and Advanced Learning (VAIAL), Vellore Institute of Technology, Vellore, Tamil Nadu, India

**Keywords:** AI-powered nutrition, cosine similarity, culturally relevant diet, explainable AI, graph retrieval-augmented generation (GraphRAG), knowledge graph, large language models (LLMs), Neo4j

## Abstract

**Introduction:**

Dietary planning is essential for managing non-communicable diseases, yet many AI-based nutrition systems lack structured knowledge grounding, demographic sensitivity, and explainability. These limitations are particularly evident in culturally diverse contexts such as India, where standard approaches often fail to align clinical dietary requirements with traditional meal patterns.

**Methods:**

This study proposes a graph-centric decision-support framework using a Graph Retrieval-Augmented Generation (Graph-RAG) architecture. A Neo4j knowledge graph models relationships among diseases, nutrients, food items, and demographic-specific Recommended Dietary Allowances (RDA). A semantic Extract, Transform, Load (ETL) pipeline integrates heterogeneous datasets and resolves terminology inconsistencies using embedding-based alignment. At inference time, nutrient requirements are retrieved from the graph and matched with food composition profiles using a deterministic cosine similarity-based ranking algorithm that prioritizes proportional nutrient balance. A language model is restricted to formatting graph-validated outputs.

**Results:**

The framework was evaluated across multiple case-study scenarios, including anemia, hypertension, and diabetes, using diverse user profiles. Results indicate improved ranking consistency, greater alignment with nutrient requirements, reduced nutrient-dominance bias, and enhanced demographic sensitivity compared with baseline approaches. Ablation analysis shows that RDA-based normalization significantly improves nutritional balance.

**Discussion:**

The results suggest that combining graph-native reasoning with constrained language generation supports transparent and knowledge-guided nutrition recommendations. The system improves interpretability while reducing risks associated with unconstrained generative models. However, the evaluation is conducted in a controlled setting, and the framework should be considered a decision-support tool rather than a clinically validated system. Future work includes uncertainty modeling, dataset expansion, and expert validation.

## 1 Introduction

Dietary patterns play a central role in the prevention and management of chronic diseases, including non-communicable diseases, by directly influencing metabolic health, immune function, and long-term wellbeing. Inadequate or imbalanced nutrient intake is strongly associated with conditions such as diabetes, hypertension, obesity, cardiovascular disease, and chronic kidney disorders. Consequently, personalized dietary planning has emerged as a critical component of preventive and therapeutic healthcare, particularly in populations experiencing rapid lifestyle transitions.

In the Indian context, dietary practices are deeply shaped by cultural traditions, regional diversity, and climatic conditions. A canonical example is the Indian Thali, which brings together cereals, pulses, vegetables, dairy, fats, and condiments into a single meal intended to provide nutritional balance. While conceptually well-founded, contemporary dietary habits have deviated from this balance, often leading to excessive carbohydrate intake, limited protein diversity, and insufficient micronutrient intake. These shifts, combined with rising disease prevalence and heterogeneous individual health profiles, challenge the effectiveness of traditional dietary norms when applied uniformly across populations. India currently has over 101 million diabetics and 136 million prediabetics, according to the Indian Council of Medical Research (ICMR) 2023 studies.

Recent advances in AI-driven nutrition recommendation systems aim to address personalization by leveraging machine learning and Large Language Models (LLMs). However, most existing systems are designed around Western dietary datasets, flat nutrient representations, or generic food items, making them poorly suited to the structural complexity of Indian meals. In particular, current approaches struggle to model multi-ingredient dishes such as the Indian Thali, where nutritional properties emerge from interactions among ingredients, preparation methods, and portion combinations. Furthermore, many systems treat clinical requirements, cultural food practices, and nutrient balance as independent factors, rather than as interdependent components of a unified dietary decision process.

Another critical limitation is the growing reliance on LLMs for diet planning. While LLMs offer flexible natural language interaction, they lack intrinsic nutritional grounding and may generate recommendations that violate dietary constraints or clinical guidelines. Without explicit integration of structured nutritional knowledge, disease-specific requirements, and culturally relevant food data, such systems risk producing recommendations that are inconsistent, non-verifiable, or clinically unsafe.

This work addresses these challenges by proposing a graph-native, knowledge-driven framework for personalized dietary planning in the Indian context. By explicitly modeling relationships among symptoms, diseases, nutrients, food ingredients, dietary guidelines, and traditional meal structures, the proposed approach aims to support clinical knowledge representations, culturally grounded, and explainable diet recommendations while minimizing reliance on unconstrained generative reasoning. Crucially, this framework is designed to seamlessly accommodate the full spectrum of dietary preferences prevalent in the Indian demographic, supporting precise nutritional optimization across vegetarian, non-vegetarian, and vegan dietary profiles.

Planning balanced diets for individuals with chronic conditions remains a challenging task for clinicians, dietitians, and public health practitioners. Decisions often require balancing multiple nutrient requirements, preventing both deficiencies and excesses, and adjusting intake based on age, gender, symptoms, and co-existing medical conditions. These considerations become even more complex in culturally diverse settings such as India, where dietary habits, ingredient availability, and traditional meal structures vary widely across regions. Existing AI systems rarely account for this diversity and tend to produce generic recommendations that overlook clinical constraints. There is a clear need for decision support tools that can translate clinical requirements into culturally appropriate meal plans while supporting transparency, traceability, and safety in the recommendation process.

In this context, a structured approach grounded in clinical knowledge representations is essential. A decision support system built on a curated knowledge representation can help practitioners understand why certain foods are recommended, how nutrient targets are met, and where trade offs occur. Such a system should not only generate personalized meal plans but also make its reasoning traceable, so that healthcare providers can verify the nutritional logic behind each recommendation. This motivates the development of a graph-based framework that brings together clinical guidelines, nutrient composition data, and demographic factors in a transparent and interpretable manner.

This study is guided by the following research questions:

- How can disease-specific nutritional requirements be systematically linked to culturally rich Indian meals such as the Thali?
- Can graph-native computation enable more reliable nutritional balance assessment than feature-based or LLM-centric approaches?
- How can Graph Retrieval Augmented Generation (GraphRAG) be constrained to support hallucination-safe, nutritionally valid dietary recommendations?

The major contributions of this paper are:

1. This work constructs a unified nutrition knowledge graph that links symptoms, diseases, nutrients, food items, and demographic-specific Recommended Dietary Allowances (RDAs) targets, providing a structured foundation of GraphRAG for transparent, nutritionally grounded dietary decision support.
2. It introduces a graph-based retrieval and ranking method in which nutrient reasoning occurs entirely within the knowledge graph. A deterministic cosine-similarity formulation is used to prioritize nutritional balance, resulting in recommendations that are both interpretable and sensitive to age and gender differences.
3. It indicates, through representative case studies, that the system produces stable and coherent meal plans while avoiding the common pitfalls of generic or nutrient-dominant recommendations. The language model is restricted to formatting validated graph outputs, supporting safe and explainable presentation of results.

The rest of the paper is organized as follows: Section 2 presents the literature review and Section 3 outlines the methodology. The experimental setup is given in Section 4, and comparative analysis is presented in Section 5. Section 6 presents the behavioral and sensitivity analysis, and the study's discussion and implications are discussed in Section 7. Section 8 addresses the limitations of the proposed system. The last section concludes the study.

## 2 Literature review

### 2.1 AI-driven diet and nutrition recommendation systems

AI has been extensively investigated as a tool for personalized diet planning and nutrition recommendations. Early work in this area largely adapted paradigms from classical recommendation systems, including content-based filtering, collaborative filtering, and hybrid models. These systems are primarily designed to align dietary recommendations with user preferences and historical consumption patterns. However, systematic analyses indicate that such approaches often prioritize palatability and user engagement over nutritional adequacy, with health constraints incorporated only as secondary filters rather than as core decision criteria (Bondevik et al., 2024).

Recent reviews highlight a shift toward more sophisticated deep learning architectures. For instance, Liu et al. (2025) demonstrate that while deep learning models have improved food recognition, they often lack the explainability required for clinical adoption. Furthermore, a 2025 systematic review found that although AI-driven interventions show promise, most existing systems function as “black boxes” (Wang et al., 2025). The reliance on static datasets also limits these systems; traditional regression models often fail to capture the complex, non-linear progression of metabolic diseases over time (Zhang et al., 2020).

### 2.2 Health-aware and constraint-based systems

To address these limitations, health-aware diet recommendation systems have incorporated machine learning models to enforce disease-specific constraints. Evwiekpaefe et al. (2023) proposed a framework for individuals with diabetes and hypertension, where classifiers restrict meal choices based on sodium thresholds. Beyond simple filtering, predictive modeling has become central to preventative nutrition. Guo et al. (2024) employ gradient-boosting models to predict vitamin D deficiency using lifestyle variables. Similarly, recent work by Lee et al. (2025) utilizes adaptive machine learning to identify key predictors of Type 2 Diabetes among older adults. In a related study, Sun et al. (2025) applied unsupervised learning to identify dietary patterns associated with obesity and diabetes risk, arguing that AI systems must move beyond single-nutrient constraints to model holistic dietary patterns.

### 2.3 Hybrid AI, LLMs, and GraphRAG frameworks

The emergence of Hybrid AI systems has introduced new paradigms for unifying the linguistic capabilities of LLMs with the structural integrity of knowledge graphs. Pan et al. (2024) provide a roadmap for these synergized systems, noting that KGs provide the factual grounding that black-box LLMs lack. A significant milestone is the development of GraphRAG, which utilizes graph-based indexing to perform global reasoning over complex datasets (Edge et al., 2024). Naganawa et al. (2025) further demonstrate that GraphRAG-enhanced systems significantly improve context relevance in real-world decision-making compared to naive vector-based RAG.

While standalone LLMs are prone to hallucination, RAG architectures mitigate these risks. Ase et al. (2025) highlight that LLMs require structured validation to reach the accuracy required for clinical dietetics. Parameswaran et al. (2025) demonstrate that RAG-enhanced systems provide more guideline-adherent nutrition advice than unguided LLMs. Expanding on this, Zhou et al. (2026) introduced NutriRAG, finding that structured data retrieval improves accuracy compared to standard LLM approaches. Miao et al. (2024) similarly showed that clinical RAG integration in nephrology significantly reduced hallucinations when answering complex patient queries about renal diets.

### 2.4 Knowledge graph reasoning in biomedicine and nutrition

Knowledge graphs enable explicit encoding of semantic relationships among foods, nutrients, and medical conditions. Chen et al. (2023) propose a health-aware food recommendation framework based on a collaborative recipe knowledge graph combined with multi-task learning. Similarly, Ma et al. (2024) introduce a nutrition-related knowledge graph neural network that applies graph convolutional networks over a structured graph linking recipes and nutrient profiles. Kobayashi et al. (2025) developed a logic-based system that uses probabilistic programming over a knowledge graph to manage lifestyle-related diseases. For clinical contexts where traceability is essential, Lodhi et al. (2023) present a knowledge-graph-driven methodology using an ontology to encode patient attributes.

The proposed work builds on advanced biomedical reasoning frameworks. BioPathNet enable multi-hop inference across deep graph structures to identify disease-symptom linkages Feng et al., (2025). Similarly, Omar and Mohammed (2025) propose a hybrid LLM-KG framework that translates natural language into executable queries to retrieve medical evidence from graphs. The complexity of high-hop retrieval is further addressed by LogosKG, which optimizes traversal for diagnostic evidence propagation (Cheng et al., 2026). While these systems primarily model drug-disease interactions, the proposed framework extends these capabilities to nutritional biochemistry. Recent research has explored various computational approaches for modeling complex relational systems and enabling data-driven decision support in biological and food domains. Graph-based analytical frameworks have been widely used to understand the structure and evolution of complex networks. For instance, Sefer (2022) proposed BioCode, a data-driven framework for learning the structural growth of biological networks by analyzing evolving connectivity patterns. Earlier work by Sefer and Kingsford (2016) introduced diffusion archaeology, a method for reconstructing the progression history of diffusion processes in networks by analyzing how information propagates across interconnected nodes. These studies demonstrate the effectiveness of network-based representations for capturing complex relational dependencies and inferring hidden propagation patterns in structured data.

In the food domain, semantic recommender systems and digital food infrastructures have increasingly been explored to support personalized and sustainable dietary choices. Sedrakyan et al. (2023) proposed a human-centric semantic recommender framework that integrates conceptual modeling, behavioral insights, and Industry 5.0 principles to promote sustainable food consumption. Complementing these recommender approaches, Gavai et al. (2025) reviewed privacy-preserving data platforms for agricultural and food supply chains, highlighting techniques such as federated learning, differential privacy, and secure multi-party computation for enabling collaborative data-driven analysis while protecting sensitive agricultural data. These developments emphasize the growing importance of robust data infrastructures and AI-enabled platforms in modern agri-food systems.

Probabilistic graphical models have also been applied to analyze risks and vulnerabilities within food supply chains. For example, Bouzembrak et al. (2024) developed a data-driven food fraud vulnerability assessment framework using Bayesian networks and failure modes and effects analysis to model complex interactions among risk factors in spice supply chains.

### 2.5 Human-centered and context-aware AI

The shift toward Human-Centered AI (HCAI) emphasizes systems that are transparent and aligned with human values. Nizamani et al. (2025) argue that context-aware systems in healthcare must move beyond simple personalization to empower patient autonomy through interpretable reasoning. This is critical in nutrition, where adherence is dependent on cultural context. Unlike prior systems that model user behavioral history, the proposed framework models explicit demographic constraints (age, gender, and clinical status) as the primary contextual drivers.

### 2.6 Demographic and cultural considerations

A persistent limitation across nutrition recommendation systems is insufficient consideration of cultural variability. Authoritative national resources such as the Indian Food Composition Tables (IFCT) (Longvah et al., 2017) and the Indian Nutrient Databank (Vijayakumar et al., 2024) provide culturally grounded nutrient baselines that differ from international standards. Recent work on domain-specific food knowledge graphs illustrates how regional diversity can be captured, like Gupta et al. (2024a), which introduced FKG.in, a knowledge graph modeling Indian recipes and ingredients. Subsequent extensions incorporate automated food composition analysis, enabling verifiable nutritional reasoning grounded in structured evidence (Gupta et al., 2024b).

While prior work demonstrates the effectiveness of probabilistic models, semantic recommenders, and data-driven platforms in food systems, these approaches exhibit different trade-offs. Probabilistic models capture uncertainty but often lack interpretability, whereas semantic recommender systems emphasize user context but may not enforce strict domain constraints. In contrast, the proposed framework adopts a deterministic knowledge-graph-driven approach that prioritizes interpretability and constraint satisfaction. However, this comes at the cost of limited uncertainty representation and reliance on explicitly structured knowledge. Thus, the contribution should be viewed as a complementary design choice within the broader landscape of AI-based food recommendation systems rather than a replacement for existing paradigms.

This comparison given in Table 1 highlights that the proposed framework should be interpreted as a complementary design choice that prioritizes interpretability and structured reasoning, rather than a replacement for probabilistic or data-driven approaches that better capture uncertainty.

**Table 1:** Comparative analysis of major AI paradigms for nutrition recommendation systems.

Approach	Strengths	Limitations	Representative works
Probabilistic models	Effectively capture uncertainty, variability, and complex dependencies in dietary and biological systems	Limited interpretability; often difficult to trace reasoning; computationally intensive in large-scale settings	Bayesian food risk modeling (Bouzembrak et al., 2024), probabilistic KG reasoning (Kobayashi et al., 2025)
Traditional recommender systems (CBF/CF/Hybrid)	Leverage user preferences and historical patterns; well-established and scalable	Often prioritize user preferences over clinical correctness; weak integration of medical constraints	Systematic review of diet recommenders (Bondevik et al., 2024), constraint-based systems (Evwiekpaefe et al., 2023)
Deep learning models	Capture complex, non-linear relationships in dietary and health data; strong predictive performance	Lack explainability (“black-box” nature); require large datasets; limited clinical trust	Deep learning for food systems (Liu et al., 2025), AI review (Wang et al., 2025)
LLM-based systems (Zero-shot/generative)	Flexible natural language interaction; adaptable to diverse user queries	Prone to hallucination; lack grounding in nutritional constraints; limited reliability in clinical contexts	LLM evaluation in nutrition (Ase et al., 2025), guideline adherence (Parameswaran et al., 2025)
RAG-based systems	Improve factual grounding by integrating external knowledge; reduce hallucination compared to standalone LLMs	Still depend on retrieval quality; may lack structured reasoning over domain-specific relationships	GraphRAG and retrieval frameworks (Edge et al., 2024; Zhou et al., 2026; Miao et al., 2024)
Knowledge graph-based systems	Provide structured, explainable reasoning; enable multi-hop relational inference; high traceability	Depend on completeness and quality of structured knowledge; limited handling of uncertainty	Health-aware KG recommenders (Chen et al., 2023; Ma et al., 2024; Lodhi et al., 2023)
Proposed GraphRAG (Deterministic KG + Constrained LLM)	Combines interpretability, structured reasoning, and reduced hallucination; ensures reproducibility and demographic sensitivity	Limited uncertainty modeling; relies on explicit knowledge representation; evaluation currently constrained to controlled settings	Proposed work

### 2.7 Research gaps

While these approaches demonstrate the potential of graph-based modeling, semantic systems, and AI-driven analytics in food-related applications, many existing methods either focus on conceptual recommender design, probabilistic risk modeling, or secure data infrastructures independently. Despite growing interest in AI-driven nutrition recommendation systems, several important gaps remain, particularly for personalized dietary planning in the Indian context.

First, while authentic resources such as the IFCT and the Indian Nutrient Databank provide detailed nutritional values, they are not always systematically linked to disease-specific nutrient requirements or symptom-level health indicators. This gap may limit the ability of existing systems to move beyond generic recommendations toward knowledge-grounded and precise dietary guidance.

Second, most current approaches appear to treat clinical nutrition requirements and culturally relevant meal planning as independent concerns. Health-aware models typically enforce medical constraints through threshold-based filtering, whereas culturally oriented systems focus on regional preferences without embedding formal nutritional reasoning. As a result, personalized diet plans may occasionally lack a balance between medical appropriateness and cultural relevance.

Finally, although GraphRAG and Hybrid AI frameworks have shown promise for structured reasoning, their application to the specific structural complexities of Indian cuisine remains limited. In particular, few systems appear to employ graph-native, deterministic reasoning to model relationships among symptoms, diseases, nutrients, ingredients, and regional recipes, or use generative models in a strictly controlled, nutritionally grounded manner.

These gaps indicate a need for an integrated, graph-first framework that connects disease-specific nutritional requirements with structured representations of Indian food systems, supporting explainable, culturally grounded, and meaningful dietary recommendations.

Motivated by these limitations, the present study proposes a knowledge-driven GraphRAG framework that integrates structured knowledge graphs with LLMs to support explainable and personalized nutrition recommendations. By explicitly modeling relationships among diseases, nutrients, food items, and dietary guidelines within a knowledge graph, the proposed approach enables transparent reasoning and culturally relevant dietary planning beyond traditional recommender or predictive models.

## 3 Methodology

The system is intended as a public health decision-support tool rather than an autonomous diagnostic system.

### 3.1 Problem formulation and notation

The personalized nutrition recommendation task is modeled as a retrieval and ranking problem within a knowledge graph G=(V,E).

Let *U* represent a user profile tuple *U* = (*A*_*demo*_, *I*_*query*_), where *A*_*demo*_ denotes demographic attributes (age and gender) and *I*_*query*_ represents the user's natural language input (e.g., stated symptoms or disease names).

#### 3.1.1 Semantic symptom-to-disease mapping

To address the ambiguity of natural language inputs, we define a semantic mapping function Φ that projects user input onto the graph's disease space. Let **e**(*text*) be a vector embedding function (via SentenceTransformers). The clinical condition *D* is identified as the canonical node $v\in V_{disease}$ that maximizes semantic similarity with the input:


$$\begin{array}{l} D=\underset{v\in V_{disease}}{\mathrm{argmax}}\left(\cos\left(\text{e}\left(I_{query}\right),\text{e}\left(v_{label}\right)\right)\right) \end{array}$$ (1)

where the embedding function maps input disease or symptom descriptions into a vector representation using a pre-trained SentenceTransformer model.

In this framework, *D* represents the primary canonical disease node (e.g., mapping high sugar levels → *Diabetes Type 2*). While the formulation theoretically supports finite multi-label sets *D* = {*d*_1_, *d*_2_, ...} for comorbidities, this study focuses on optimizing ranking for the primary identified condition.

#### 3.1.2 Ranking formulation

The objective is to retrieve a set of foods $F=\left\{f_{1},f_{2},...,f_{n}\right\}$ from G and rank them based on alignment with the nutritional constraints of *D*. We define two vectors in the high-dimensional nutrient space ℝ^*k*^, where *k* is the number of nutritionally aligned nutrients for *D*:

- **Target vector (V_*target*_):** Represents the ideal nutrient intake derived from RDA standards adjusted for *A*_*demo*_.
- **Candidate vector (V_*f*_):** Represents the nutritional composition of a candidate food item *f*, normalized against the RDA caps.

The ranking score *S*(*f, U*) is computed as the cosine similarity between **V**_*target*_ and **V**_*f*_.

### 3.2 System architecture

**Figure 1:**
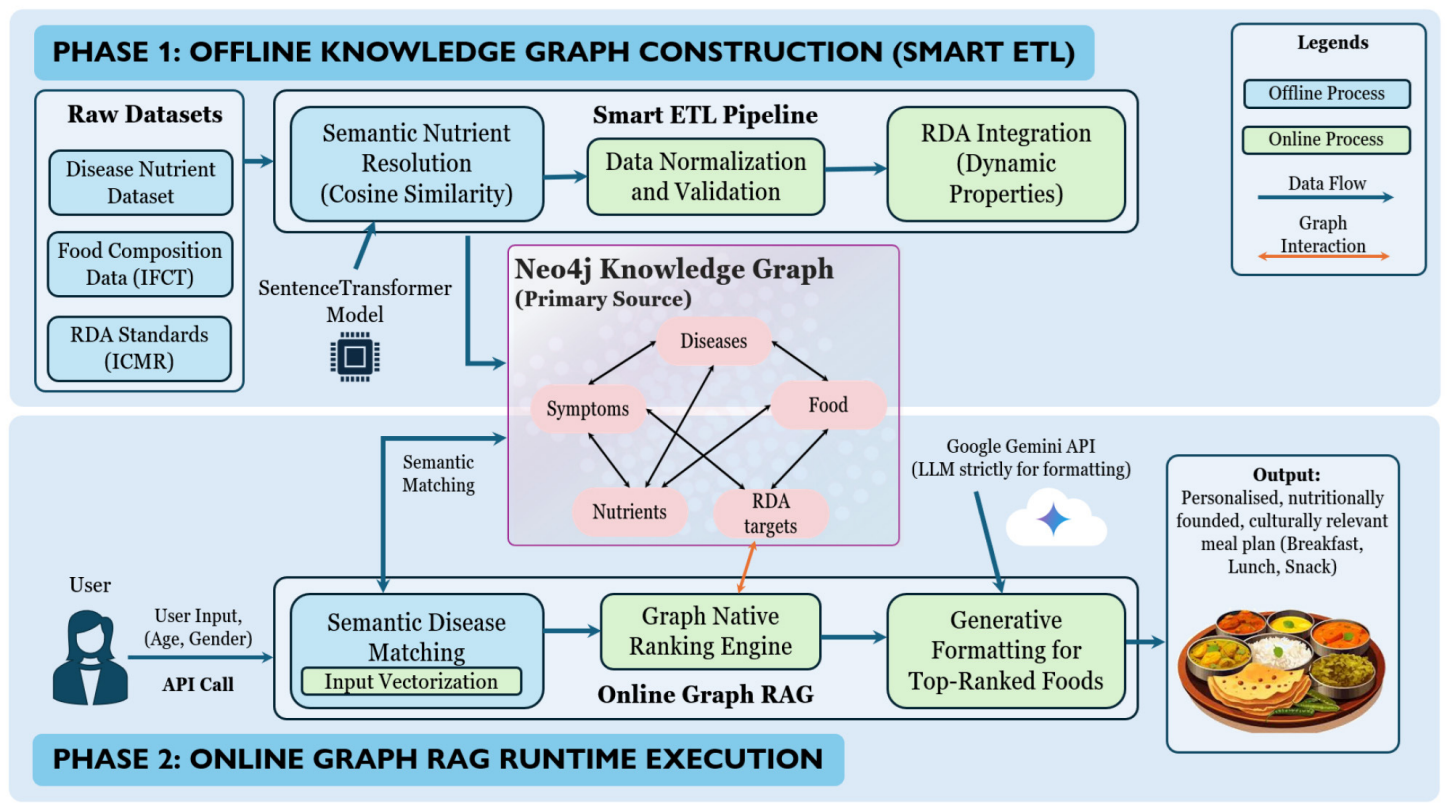
Overview of the proposed GraphRAG framework. Phase 1 performs offline knowledge graph construction through a semantic ETL pipeline integrating symptoms, disease, nutrient, food, and RDA datasets. Phase 2 executes online personalized recommendation using graph-native retrieval and ranking, followed by constrained language generation for presentation. All nutritional reasoning is performed within the knowledge graph, while the LLM is restricted to formatting validated outputs.

The system is designed as a GraphRAG framework, as shown in Figure 1.

Unlike traditional systems that rely on flat-file lookups, this architecture centers on a Neo4j knowledge graph that serves as the single source of truth for disease-nutrient-food relationships. Neo4j is used to enable native multi-hop traversal and in-database vector computations, avoiding lossy feature flattening. This helps in the explainability of the decision made by the system, and can aid in decision-making of dietary plans. The workflow operates in two distinct phases:

**Phase 1: Knowledge graph construction (semantic ETL)** To ensure data integrity, a semantic Extract, Transform, Load (ETL) pipeline is employed to ingest raw datasets into the graph structure.

- **Semantic nutrient resolution:** The ETL engine utilizes a SentenceTransformer model to compute embedding vectors for clinical nutrient terms. It maps them to scientific nutrient nodes in the graph using a cosine similarity threshold (τ > 0.5). The threshold was empirically chosen to balance recall and semantic precision during vocabulary alignment. This resolves vocabulary mismatches [e.g., mapping potassium to Potassium (K)] before data commitment.
- **RDA integration:** A critical innovation is the integration of official RDA values directly into the graph schema. RDA targets are stored as dynamic properties on nutrient nodes, keyed by age and gender identifiers, enabling efficient runtime retrieval [*O*(1) complexity].

**Phase 2: Runtime GraphRAG execution** This stage activates per API call, utilizing the pre-constructed graph to deliver real-time results.

1. **Semantic disease matching:** User input is vectorized to identify the closest disease node in the graph, robustly handling natural language variations.
2. **Graph-native ranking:** The core engine executes the deterministic ranking algorithm (Algorithm 1) to retrieve the optimal set of foods.
3. **Generative formatting [natural language generation (NLG) module]:** The top-ranked, mathematically validated foods are passed to the Google Gemini API. Unlike standard LLM approaches, the model is strictly constrained to *formatting* duties—converting the structured graph data into a culturally relevant 1-day Indian Thali meal plan without adding hallucinated food items.

### 3.3 Graph-native ranking algorithm

The core computational engine replaces heuristic filtering with a deterministic vector-based ranking strategy. All retrieval and vector computations are executed directly within the Neo4j database using graph-native queries, avoiding external feature materialization. The procedure is formally defined in Algorithm 1, and the logical execution steps are detailed below.

Algorithm 1 Graph-native cosine similarity ranking. 
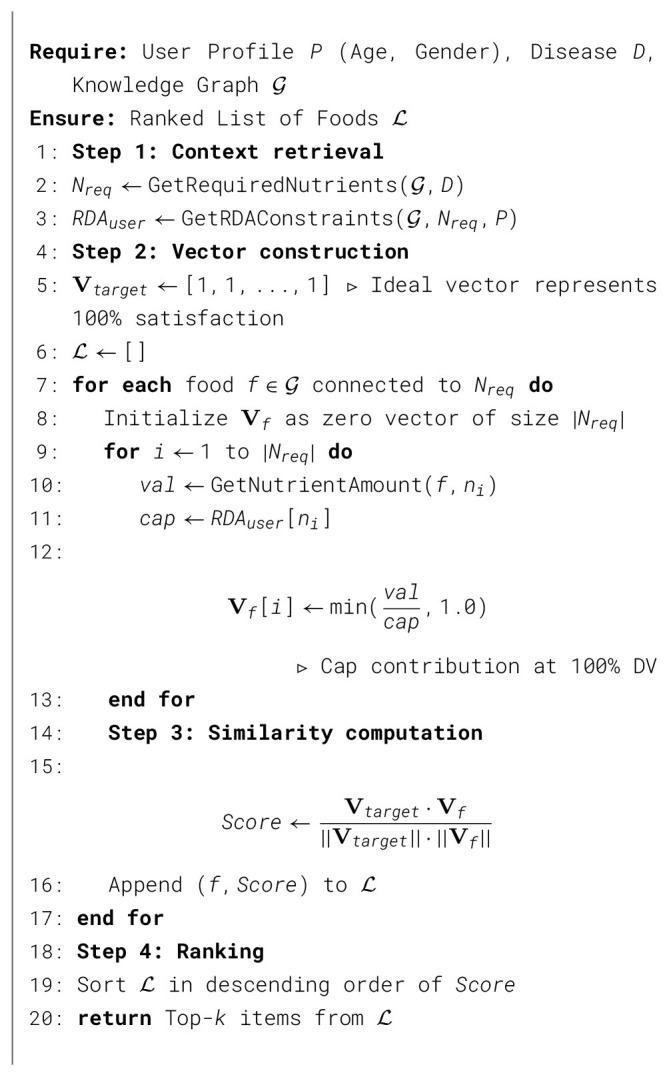

#### Algorithmic walkthrough

The algorithm operates in four sequential stages to ensure that recommendations are not merely nutrient-rich but structurally balanced according to the user's specific pathology:

**Step 1: Context retrieval** Unlike static vector searches, the system first dynamically traverses the knowledge graph starting from the identified disease node *D*. It retrieves the set of clinically required nutrients *N*_*req*_ (e.g., for Anemia: Iron, Vitamin C, and Folic Acid) via [:REQUIRES] edges. Simultaneously, it queries the specific RDA constraints (*RDA*_*user*_) encoded on the nutrient nodes, selected based on the user's age and gender attributes *P*.

**Step 2: Vector construction and normalization** For every candidate food item *f* connected to the required nutrients, the system constructs a dense nutrient vector **V**_*f*_. A critical novelty here is the **RDA-capping mechanism**. Raw nutrient values are normalized against the user's specific RDA cap.


$$\begin{array}{l} {\text{V}}_{f}\left[i\right]=\min\left(\frac{{\text{Amount}}_{f,i}}{{\text{RDA}}_{user,i}},1.0\right) \end{array}$$ (2)

This capping ensures that a food providing 500% of the daily Iron requirement does not skew the ranking score. It forces the algorithm to penalize “superfoods” that are rich in only one dimension, promoting items that contribute to a broader set of requirements.

**Step 3: Cosine similarity scoring** The system defines an “Ideal Target Vector” **V**_*target*_ as a unit vector where every dimension equals 1.0 (representing 100% satisfaction of all required nutrients). The ranking score is computed as the cosine similarity between the food vector **V**_*f*_ and this ideal target. Mathematically, this measures the *angular alignment* between the food's nutrient profile and the ideal balanced nutrient profile. A score close to 1.0 indicates that the food provides a proportional, balanced coverage of all required nutrients, rather than a simple abundance of a single nutrient. This formulation assumes equal weighting across all nutrients, treating each dimension as equally important in the similarity computation.

**Step 4: Deterministic ranking** Finally, the candidate foods are sorted by their similarity scores. This deterministic approach ensures that the top-*k* results are mathematically verifiable and consistent, eliminating the stochastic randomness often observed in pure LLM-based generation.

### 3.4 Dataset description

Together, Table 2 form the foundation of the knowledge graph, enabling the system to connect clinical conditions, nutritional requirements, and culturally relevant Indian food items within a unified reasoning framework.

**Table 2:** Datasets used for knowledge graph construction.

Dataset	Source	Purpose in the system	Graph representation
Disease–nutrient mapping dataset	Curated from clinical nutrition guidelines (WHO, CDC, and NIH)	Links diseases and symptoms to required nutrients and dietary considerations. Enables the system to retrieve disease-specific nutrient requirements during query processing.	(:Disease)-[:REQUIRES]->(:Nutrient) relationships
Indian food composition tables (IFCT 2017)	National Institute of Nutrition (India)	Provides nutritional composition of 542 Indian food items including macronutrients, micronutrients, and bioactive compounds. Used to construct food–nutrient relationships.	(:Food)-[:CONTAINS_NUTRIENT]->(:Nutrient) relationships
Indian recommended dietary allowances (ICMR-NIN 2020)	Indian Council of Medical Research (ICMR)	Supplies age- and gender-specific nutrient intake standards used to construct personalized nutrient target vectors for ranking food items.	Dynamic RDA properties on (:Nutrient) nodes
Nutrient abbreviation and semantic mapping dataset	Curated mapping dictionary from IFCT schema	Resolves inconsistencies in nutrient naming (e.g., technical dataset codes vs. scientific nutrient names) and supports semantic normalization during the ETL process.	Used during ETL for schema normalization

To construct the knowledge graph, the system integrates four primary datasets. The resulting knowledge graph comprises 827 nodes: 70 diseases, 542 unique Indian food items (IFCT 2017), and 215 nutritionally aligned macro- and micronutrients. It contains approximately 58,000+ total relationships, primarily [:CONTAINS_NUTRIENT] and [:REQUIRES] edges. Each dataset serves a distinct role in the graph schema, linking clinical pathology to nutritional biochemistry. These datasets help ground the system's decisions in nutritional biochemistry and trace the reasoning behind each recommendation. The knowledge graph schema and entity relationships are provided in Table 3.

Table 3Knowledge graph schema and entity relationships.

**Table:** 

Entity type	Node label	Description	Example
Disease	:Disease	Represents medical conditions associated with specific nutritional requirements.	Anemia, Hypertension, Diabetes
Nutrient	:Nutrient	Represents macro and micronutrients required for human health. Stores demographic-specific RDA values as node properties.	Iron, Vitamin C, Zinc
Food Item	:Food	Represents individual food ingredients or items derived from the IFCT dataset. Each node stores nutritional composition per 100g.	Spinach, Bajra, Paneer

**Table:** 

Relationship	Schema	Description
Disease–nutrient relationship	(:Disease)-[:REQUIRES]->(:Nutrient)	Encodes the nutritional requirements or dietary constraints associated with a specific disease condition.
Food–nutrient relationship	(:Food)-[:CONTAINS_NUTRIENT]->(:Nutrient)	Links food items to the nutrients they contain along with their quantity values.
Symptom–disease relationship	(:Symptom)-[:INDICATES]->(:Disease)	Supports semantic mapping of user-reported symptoms to clinical disease nodes during query processing.

#### Disease-nutrient mapping dataset

**Figure 2:**
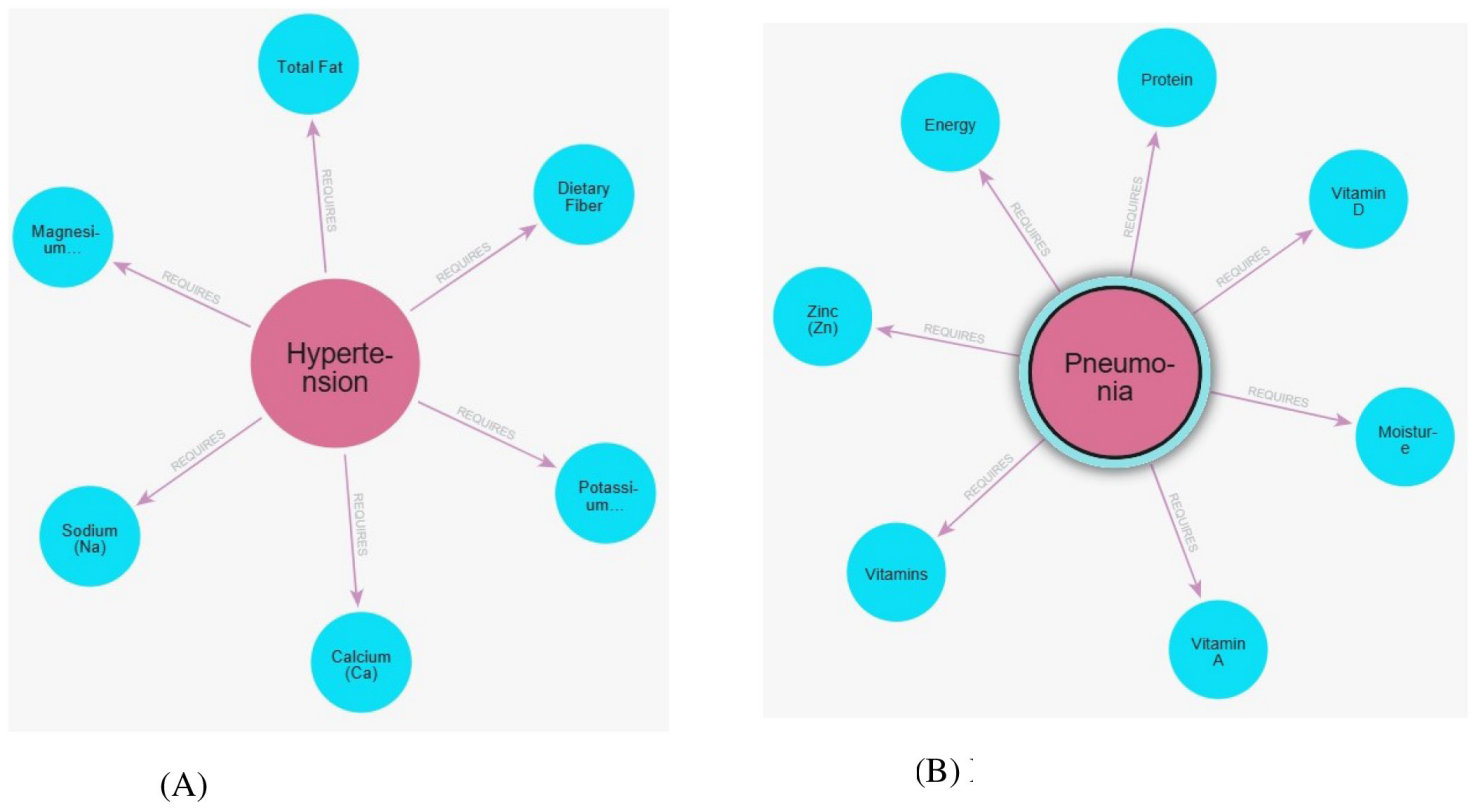
Example subgraphs showing disease–nutrition relationship for hypertension and pneumonia within the knowledge graph. **(A)** Hypertension. **(B)** Pneumonia.

This dataset serves as the clinical grounding for the system. It maps over 70 distinct disease conditions to their specific nutritional requirements, contraindications, and clinical notes. The data was curated from medical guidelines (WHO, CDC, NIH) to ensure accuracy; a sample is shown in Table 4. It creates (:Disease)-[:REQUIRES]->(:Nutrient) relationships in the graph. Simple example graphs are given in Figure 2.

**Table 4:** Sample records from the Disease-nutrient mapping dataset.

Disease	Recommended nutrients	Clinical notes
Tonsillitis	Fluids, Protein, Vitamin C, Zinc	Emphasis on soft, soothing foods for recovery.
Common Cold	Vitamin C, Zinc, Protein, Vitamin D	Supportive nutrition to maintain hydration.
Anemia	Iron, Vitamin C, Folic Acid	Vitamin C is required to enhance Iron absorption.

**Figure 3:**
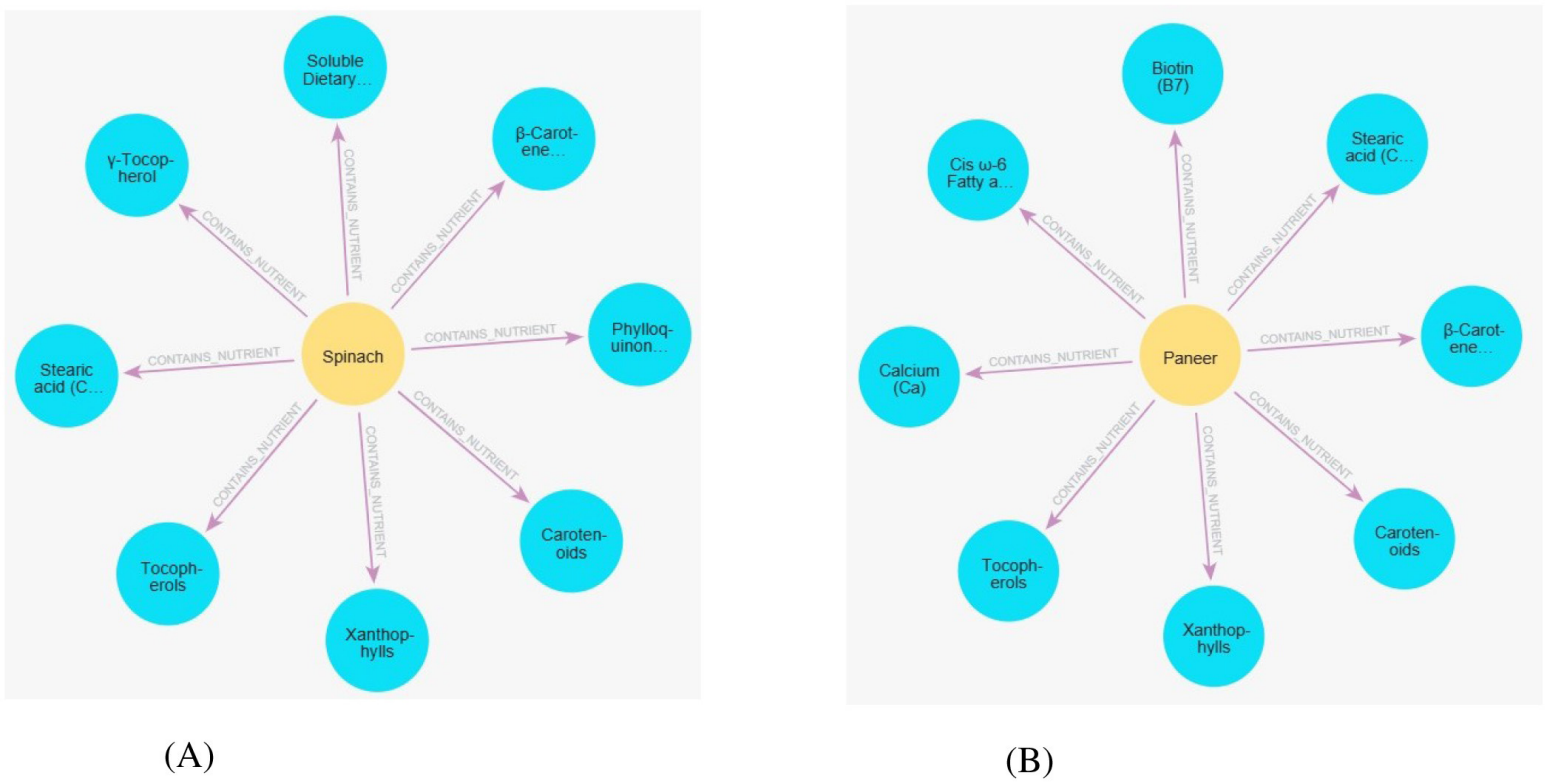
Example subgraphs showing food–nutrient relationships for spinach and paneer within the knowledge graph. **(A)** Spinach. **(B)** Paneer.

**IFCT 2017:** This dataset provides the nutritional backbone of the system. It contains the detailed biochemical breakdown of 542 native Indian foods (per 100g edible portion), covering over 215 nutrients including macronutrients, micronutrients, and bioactive compounds. A sample of the dataset is given in Table 5. Unlike generic Western datasets, this includes region-specific foods. It is used to populate (:Food) nodes and [:CONTAINS_NUTRIENT] edges in the graph. Sample graphs for spinach and paneer are shown in Figure 3.

**Table 5:** Sample nutritional profiles from the IFCT dataset (per 100g).

Code	Food name	Energy (kJ)	Protein (g)	Iron (mg)	Fiber (g)
A001	Amaranth seed, black	1,490	14.59	5.76	14.59
A002	Amaranth seed, pale	1,489	13.27	5.80	13.27
A003	Bajra (Pearl Millet)	1,456	10.96	6.42	11.49

#### Indian RDA Standards (ICMR 2020)

To enable personalization, the system incorporates the official “Nutrient Requirements for Indians” (ICMR-NIN 2020). This dataset specifies the RDA for 30+ nutrients across different age groups, genders, and activity levels. It is used in the graph to store as dynamic properties on (:Nutrient) nodes (e.g., rda_male_19_30). Table 6 shows examples of the dataset.

**Table 6:** Demographic-specific RDA constraints used for logic rules.

Nutrient	Group	Age	Daily requirement
Iron	Men	19–30	19 mg
Iron	Women	19–30	29 mg
Protein	Men	19–30	54 g

#### Nutrient abbreviation and semantic map

Raw datasets often use cryptic column headers (e.g., protcnt, fibtg). This dataset acts as a semantic dictionary, mapping technical codes to human-readable scientific names. This is critical for the Smart ETL engine to correctly ingest and link data. It is used during the ETL phase to normalize schema keys. Table 7 provides a sample of the mapping.

**Table 7:** Mapping codes to semantic labels for graph normalization.

Code	Scientific name	Tags/keywords
protcnt	Protein	Total essential proximate macronutrient
fibtg	Dietary fiber	Total fiber roughage bulk indigestible
facn3	Cis ω-3 Fatty acids	Omega 3 healthy fat

### 3.5 Implementation details

To ensure reproducibility, the Smart ETL pipeline and the execution environment are configured as follows:

- **Preprocessing & normalization:** Raw data from the IFCT and ICMR datasets undergo a multi-step normalization. Nutrient units are standardized to a common scale (e.g., mg or mcg) to prevent magnitude bias during vector construction. Missing values in food composition are handled using a k-Nearest Neighbor (k-NN) imputation based on food category averages.
- **Synonym mapping:** We employ the all-MiniLM-L6-v2 SentenceTransformer model to resolve terminology mismatches. This allows the system to semantically map colloquial symptoms (e.g., feeling tired) to clinical terms (e.g., Fatigue) with a high degree of accuracy.
- **Technical stack:** The backend is built on Python 3.9 using FastAPI for request handling. The knowledge graph is hosted on Neo4j Community Edition, utilizing Cipher queries for graph-native vector arithmetic.

### 3.6 Hyperparameter configuration

Table 8 summarizes the key parameters used within the GraphRAG framework to balance retrieval recall and precision.

**Table 8:** Hyperparameter settings for the recommendation engine.

Parameter	Value	Reason/source
Embedding model	all-MiniLM-L6-v2	Optimal performance for short clinical text
Similarity threshold (τ)	0.5	Empirically determined for semantic precision
Vector dimension	384	Standard for MiniLM architecture
Top-k foods	10	Optimized for daily Thali meal structure

### 3.7 Evaluation protocol

To validate the system's performance and robustness across varied landscapes, the following protocol was established:

- **Experimental scope:** The evaluation was conducted using 20 diverse user profiles, incorporating variations in age, gender, and physiological states to ensure high demographic sensitivity.
- **Disease coverage:** While the framework explicitly models over 70 distinct disease conditions, the evaluation protocol assesses the system's ranking logic across a broad spectrum of clinical scenarios to confirm that the retrieval and capping mechanisms remain consistent regardless of the specific pathology.
- **Statistical aggregation:** To provide a transparent overview of system efficacy, performance metrics are averaged across all experimental trials, ensuring that the reported results reflect the generalizable stability of the GraphRAG architecture.

## 4 Experimental setup

This evaluation is designed as a controlled, proof-of-concept demonstration of the proposed framework. The relevance criteria and user profiles are constructed based on domain-informed nutritional constraints within the knowledge graph. Therefore, the results primarily demonstrate internal consistency and system behavior rather than externally validated clinical effectiveness.

This section evaluates the performance of the proposed GraphRAG framework. It is important to note that, at present, no standardized benchmarks exist for disease-aware, culturally specific nutrition recommendation systems, particularly for the Indian dietary context. Consequently, this study establishes a comparative evaluation framework using standard retrieval metrics alongside domain-specific nutritional constraints to assess system efficacy.

### 4.1 Experimental setup

The system was evaluated on twenty diverse user profiles spanning variations in age, gender, and health conditions. While the constructed knowledge graph supports over 70 diseases, experimental validation focused on three high-impact chronic conditions—*Anemia, Hypertension, and Diabetes*—selected due to their prevalence and distinct nutritional requirements.

The backend execution, including graph queries and ranking logic, was performed on a local machine using Neo4j and Python-based inference modules. Generative response formatting was handled via the Google Gemini cloud API.

#### 4.1.1 Latency note

While the core graph retrieval phase is consistently executed in **under 1.2 seconds** (demonstrating real-time capability), the total end-to-end latency averaged **29.7 seconds** due to the external processing overhead of the Cloud LLM API.

#### Hardware and software specifications

To ensure reproducibility, the experimental environment was configured with the following specifications:

**Hardware configuration:**

- **Processor:** Standard development machine (Intel/AMD) with multi-threading support.
- **Accelerator:** NVIDIA GPU (Recommended for local embedding computations).
- **Network:** Stable cloud API access for Google Gemini inference.

#### Software availability and requirements

- Language: Python 3.9+.
- Key libraries: pandas, torch, sentence-transformers, google.generativeai, and dotenv.
- Database: Neo4j Community Edition (Graph Native).

#### Data sources

The system uses the structured CSV datasets described in Section 3.4 (*disease_nutrients.csv, Food-nutrient_dataset.csv*, etc.). The knowledge graph schema statistics are given in Table 9.

**Table 9:** Knowledge graph schema statistics.

Graph element	Count	Description
Diseases	70	Nodes for clinical conditions (e.g., Anemia, diabetes)
Foods	542	Nodes for unique Indian food items (IFCT 2017)
Nutrients	215	Nodes for nutritionally aligned macro/micronutrients
Nodes (Total)	827	Total entities in the graph (Diseases+Foods+Nutrients)
Edges (Total)	≈58,000+	Total relationships (mainly [:CONTAINS_NUTRIENT] and [:REQUIRES])

### 4.2 Reproducibility and implementation details

The datasets utilized in this study are derived from publicly available sources, including the Indian Food Composition Tables (IFCT 2017) and ICMR-NIN RDA guidelines (2020). The knowledge graph schema, which comprises 827 nodes and approximately 58,000 relationships, is detailed in Section 3.4, Tables 9, 10.

**Table 10:** Summary of system components and evaluation setup.

Component	Description
Dataset	IFCT 2017 + ICMR-NIN 2020
Graph database	Neo4j Community Edition
Architecture	Graph-RAG (Neo4j + Gemini)
Evaluation	20 diverse user profiles
Clinical focus	3 representative conditions (anemia, hypertension, diabetes)
Primary metrics	NDCG, precision, %DV deviation, RSS

#### Reproducibility and workflow illustration

The dataset construction process involves mapping disease–nutrient relationships from clinical guidelines and integrating them with IFCT food composition data through a semantic ETL pipeline. Nutrient values are normalized and aligned with age- and gender-specific RDA targets.

At runtime, user input is first mapped to disease nodes using semantic similarity. The corresponding nutrient requirements are retrieved from the knowledge graph, and candidate food items are identified through graph traversal. These candidates are then ranked using cosine similarity between the food nutrient vectors and the ideal target vector.

A representative graph query used for retrieving candidate foods is as follows:


MATCH (d:Disease {name: $disease})-
[:REQUIRES] ->(n:Nutrient)
MATCH (f:Food)-[:CONTAINS]->(n)
RETURN f, n


The complete implementation, including graph construction scripts and query workflows, is available in the accompanying repository. All experiments reported in this study can be reproduced using the provided scripts and predefined evaluation configurations available in the repository. The dataset construction involves mapping disease–nutrient relationships from clinical guidelines and integrating them with IFCT food composition data through a semantic ETL pipeline. Example graph queries and workflow steps are provided in the repository to facilitate reproduction.

The preprocessing workflow includes unit normalization, missing value handling, and embedding-based synonym mapping using the all-MiniLM-L6-v2 model to resolve terminology mismatches. To facilitate reproducibility, the complete implementation details—including the semantic ETL pipeline (migrate_to_graph.py), the graph-native ranking engine (Algorithm 1), and the evaluation scripts—are made available in our open-source repository at https://github.com/DecoderOP/Indian-thali. The system is designed to be platform-independent and can be reproduced using a Neo4j Community Edition database and a Python 3.9+ environment.

### 4.3 Evaluation criteria

The evaluation approach in this study is based on case-driven analysis rather than large scale quantitative benchmarking. At present, no standardized datasets exist for culturally specific and clinically constrained nutrition planning, particularly for Indian dietary patterns that vary by region, preparation methods, and demographic requirements. As a result, conventional accuracy-based metrics are not applicable, because dietary recommendations are not classification outcomes but constraint satisfaction decisions involving multiple nutrients, symptoms, and age or gender specific RDA targets. In this context, case study evaluation is an established practice in decision support research, as it allows assessment of whether the system behaves consistently, respects clinical constraints, and avoids nutritionally unsafe recommendations. This method also enables examination of the system's explainability, stability, and sensitivity to demographic variations, which are central to the design goals of a transparent, coherent nutrition decision-support tool.

In this study, relevance refers to the degree to which a food item satisfies the nutrient requirements associated with a given condition, as defined within the knowledge graph. Due to the absence of standardized benchmarks for culturally specific, disease-aware nutrition planning, evaluation was conducted using a hybrid framework combining constraint-based quantitative metrics and expert qualitative validation. This approach is consistent with prior healthcare recommendation studies, where correctness is defined by clinical compliance, demographic sensitivity, and explainability rather than label prediction accuracy on static datasets.

The evaluation focuses on six specific metrics categorized across five key quantitative dimensions:

- **Standard ranking quality:** Assessing retrieval effectiveness using **NDCG@k** and **Precision@k** to provide a comparative benchmark against baseline recommendation models.
- **Rank stability (consistency):** Measuring the robustness of rankings against demographic perturbations via the **Rank stability score (RSS)**.
- **Nutrient coverage:** Assessing the breadth of essential nutrients provided by recommendations through the **Nutrient coverage score**.
- **Demographic sensitivity:** Quantifying the alignment between recommended nutrient profiles and specific RDA targets through **%DV deviation**.
- **Nutrient balance variance:** Evaluating the proportionality of nutrient distribution to help reduce superfood bias.

### 4.4 Quantitative metrics definitions

To rigorously assess the proposed framework's behavior, we define six specific metrics mapping to the five dimensions identified above. These comprise standard ranking metrics to evaluate retrieval performance and domain-specific nutritional metrics to capture domain constraints. For each metric, we provide the mathematical formulation and a comparative example to illustrate its evaluation logic.

#### Precision@k

Precision@k measures the proportion of recommended items in the top-*k* results that are nutritionally aligned to the user's profile.


$$\begin{array}{l} Precision@k=\frac{1}{k}\overset{k}{\underset{i=1}{\sum }}I\left({\text{food}}_{i}\text{is nutritionally aligned}\right) \end{array}$$ (3)

##### Definition of relevance

To operationalize this metric, nutritional relevance is defined as the degree to which a food item satisfies the specific nutrient requirements and disease-specific thresholds retrieved from the knowledge graph. Relevance labels were assigned based on whether a food item satisfied at least two of the required disease-specific nutrient thresholds defined by the knowledge graph.

**Comparative example:**

- **Scenario A (high precision):** Out of the top 5 recommended foods, 4 meet at least two primary clinical thresholds. Score = 4/5 = 0.8.
- **Scenario B (low precision):** Only 1 out of the top 5 recommended foods meets the multi-nutrient requirement. Score = 1/5 = 0.2.

*Result:* Higher precision indicates that the system supports the identification of relevant items within the immediate results.

#### NDCG@k

Normalized Discounted Cumulative Gain (NDCG) assesses the ranking quality by accounting for the position of relevant food items, where higher-ranked relevant items contribute more significantly to the final score.


$$\begin{array}{l} NDCG_{k}=\frac{\sum_{i=1}^{k}\frac{2^{rel_{i}}-1}{\log_{2}\left(i+1\right)}}{IDCG_{k}} \end{array}$$ (4)

where *IDCG*_*k*_ denotes the Ideal Discounted Cumulative Gain at rank *k*, computed by ordering items in descending order of relevance, representing the maximum achievable DCG. This normalization ensures that NDCG values lie between 0 and 1, enabling consistent comparison across different ranking scenarios.

##### Relevance mapping

In this framework, the relevance score *rel*_*i*_ is treated as a binary value {0, 1} based on the same clinical criteria used for Precision@k, satisfying at least two disease-specific nutrient requirements retrieved from the knowledge graph. This approach allows the metric to indicate whether the system successfully prioritizes medically appropriate options at the top of the Thali recommendation.

**Comparative example:**

- **Scenario A (optimal ranking):** A food item satisfying the multi-nutrient clinical threshold is placed at Rank 1.
- **Scenario B (suboptimal ranking):** The same nutritionally aligned food item is placed at Rank 5.

*Result:* The metric indicates higher performance for Scenario A, as it suggests the system successfully prioritizes the most relevant clinical options.

#### Rank stability score

Rank stability quantifies the consistency of the ranking logic when demographic variables undergo minor perturbations (e.g., Age 30 vs. Age 31), supporting system robustness rather than stochastic behavior. It is calculated using Spearman's rank correlation coefficient (ρ) between two ranked lists (*R*_*A*_, *R*_*B*_):


$$\begin{array}{l} RSS\left(R_{A},R_{B}\right)=1-\frac{6\sum d_{i}^{2}}{n\left(n^{2}-1\right)} \end{array}$$ (5)

Where *d*_*i*_ is the rank difference for food *i*.

**Comparative example:**

- **Scenario A (high stability):** User A (Age 30) and User A' (Age 31) receive rankings [*f*_1_, *f*_2_, *f*_3_] and [*f*_1_, *f*_3_, *f*_2_] respectively. The minimal swap results in an RSS ≈ 0.9, suggesting logical consistency.
- **Scenario B (instability):** If a minor age shift resulted in a completely shuffled list [*f*_3_, *f*_1_, *f*_2_], the RSS would drop to <0.5, indicating the model behavior as potentially unstable.

Metrics such as %DV deviation and nutrient coverage are domain-specific measures designed to capture nutritional alignment and are not standard information retrieval metrics.

#### %DV deviation

To quantify alignment with personalization targets, we measure the Mean Absolute Deviation (MAD) of the food's nutrient profile from the user's ideal intake (100% RDA).


$$\begin{array}{l} \text{Mean \%DV Deviation}=\frac{1}{N}\overset{N}{\underset{i=1}{\sum }}|\min\left(\%DV_{i},100\right)-100| \end{array}$$ (6)

**Comparative example:** Consider an RDA Target of 100% for Iron.

- **Food A (balanced):** Provides 90% DV of Iron. Deviation = |90 − 100| = 10.
- **Food B (deficient):** Provides 15% DV of Iron. Deviation = |15 − 100| = 85.

*Result:* The metric supports the selection of Food A (Lower Deviation), prioritizing items that bring the user closer to their daily target.

#### Nutrient coverage score

To support the requirement that top-ranked foods satisfy a broad range of clinical requirements rather than just one, we define Coverage as the fraction of required nutrients meeting a clinical threshold τ (set to 20% DV).


$$\begin{array}{l} \text{Coverage}\left(f\right)=\frac{\sum_{j=1}^{N}𝕀\left(\%DV_{j}\geq \tau \right)}{N} \end{array}$$ (7)

**Comparative Example:** Assume a disease requires three nutrients: {Iron, Vitamin C, Zinc}.

- **Food A (specialized):** Rich in Iron (50%) but low in Vitamin C (5%) and Zinc (2%). Only 1 nutrient meets the threshold. Score = 1/3 ≈ 0.33.
- **Food B (holistic):** Moderate in Iron (25%), Vitamin C (30%), and Zinc (22%). All three meet the threshold. Score = 3/3 = 1.0.

*Result:* The metric indicates better performance for food B, helping ensure the recommendation addresses the holistic disease profile.

#### Nutrient balance variance

To mathematically help reduce superfood bias, where a food dominates rankings due to a single excessive nutrient spike, we measure the variance of capped %DV contributions.


$$\begin{array}{l} \text{Variance}=\frac{1}{N}\overset{N}{\underset{i=1}{\sum }}{\left(x_{i}-\bar{x}\right)}^{2} \end{array}$$ (8)

**Comparative Example:**

- **Food A (skewed profile):** Contains 400% DV of Vitamin A (capped at 100%) but only 5% of other required nutrients. The spread between 100 and 5 creates high variance.
- **Food B (balanced profile):** Contains 50% DV across all required nutrients. The spread is minimal, creating **low variance**.

*Result:* The ranking engine penalizes high variance, helping minimize the impact of single-nutrient superfoods from overshadowing balanced meal options.

## 5 Quantitative results

This evaluation serves as a controlled, proof-of-concept demonstration of the system's capabilities. The relevance criteria and user profiles utilized are constructed based on the nutritional constraints encoded within the knowledge graph. Consequently, these results demonstrate the system's internal reasoning and ranking consistency rather than independent clinical validation.

### 5.1 End-to-end system walkthrough: a running example

To illustrate the complete operational pipeline, we trace the execution flow for a specific user scenario. This walkthrough demonstrates how the GraphRAG framework transforms unstructured input into a structured, nutritionally valid, and culturally familiar recommendation.

#### Stage 1: user input and profiling

**User persona:** A 28-year-old female, currently pregnant. **Natural language query:** “I have been feeling very dizzy lately and my doctor said my hemoglobin is low.”

The system parses this input into a structured profile tuple *U*:

- **Demographics (A_*demo*_):** {Age: 28, Gender: Female, Condition: Pregnant}
- **Input Text (I_*query*_):** “dizzy... hemoglobin low”

#### Stage 2: semantic disease mapping

The semantic mapping function Φ projects the input text onto the knowledge graph.

1. The embedding model generates a vector **v**_*input*_ for hemoglobin low.
2. It computes cosine similarity against all disease nodes.
3. **Result:** The node **Iron Deficiency Anemia** is identified as the best match (*Sim* = 0.92), triggering the retrieval of its required nutrients: {Iron, Vitamin C, Folic Acid}.

#### Stage 3: dynamic graph context retrieval

The system queries the knowledge graph for nutritional constraints specific to the user's demographic (*A*_*demo*_).

- **RDA lookup:** The graph retrieves the property rda_pregnant_woman for the required nutrients.
- **Constraints loaded:**
  - Iron target: **27 mg** (Higher than standard adult female).
  - Vitamin C target: **65 mg**.
  - Folic Acid target: **570 mcg**.

#### Stage 4: vector-based ranking (the RAG step)

The system retrieves candidate foods connected to these nutrients and ranks them using Algorithm 1. To illustrate the logic, we compare a common suggestion (*Spinach*) against a high-performance native ingredient (*Drumstick Leaves/Moringa*).

**Candidate 1: Spinach (Palak) - 100g**

- **Nutrient Profile:** Iron: 2.9 mg (≈10% DV); Vit C: 30 mg (≈46% DV); Folate: Good
- **Vector V_*spinach*_:** [0.10, 0.46, 0.25]
- **Analysis:** A balanced vector, but the magnitude is limited by moderate Iron content.

**Candidate 2: Drumstick leaves (Moringa) - 100g**

- **Nutrient Profile:** Iron: 4.56 mg (≈17% DV); Vit C: 108 mg (Capped at 1.0)
- **Vector V_*moringa*_:** [0.17, 1.0, 0.10]
- **Result:** Moringa achieves a higher cosine similarity score because its Vitamin C content is exceptionally high (>100% DV), which the knowledge graph recognizes as critical for maximizing the absorption of the non-heme iron.

#### Stage 5: generative formatting

The top-ranked structured list (including Moringa, Bajra, and Guava) is passed to the LLM (Gemini) with a strict formatting prompt to generate culturally coherent meal pairings.

**Final output presented to user:**

“Based on your pregnancy and low hemoglobin levels, here is a customized plan designed to maximize Iron absorption through natural Vitamin C synergy:

- **Breakfast: Methi (Fenugreek) thepla with curd** *Scientific Basis:* Fenugreek leaves are a potent source of Iron (**5.7 mg/100g**) and Folic Acid, essential for maternal health.
- **Lunch: Bajra Roti with drumstick leaves (Moringa) dal** *Scientific Basis:* Pearl Millet (Bajra) provides high Iron (**6.4 mg**). Pairing it with Drumstick leaves is critical as they contain **108 mg of Vitamin C** (approx. 2x daily requirement), which chemically converts non-heme iron into an absorbable form.
- **Snack: Fresh guava** *Scientific Basis:* Guava is chosen over citrus because it provides **214 mg of Vitamin C** per 100g, serving as a powerful bio-enhancer for the iron consumed earlier in the day.

**Figure 4:**
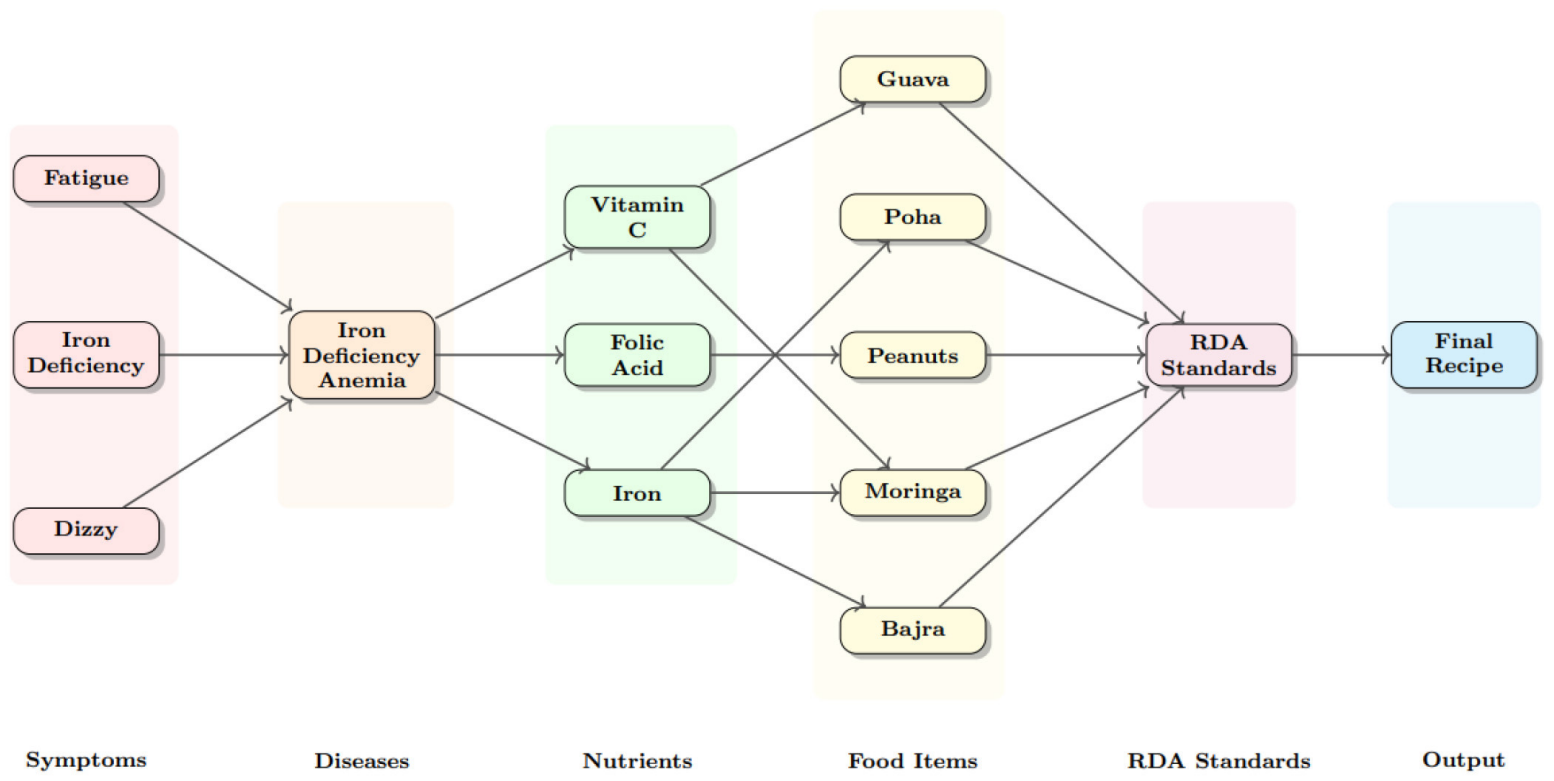
Visualization of the integrated knowledge graph structure used in the proposed Graph–RAG framework. The graph illustrates the relationships among disease nodes, nutrient nodes, and food item nodes constructed from the IFCT dataset and nutritional guidelines. Disease nodes are connected to required nutrients through REQUIRES relationships, while food items are linked to nutrients through CONTAINS_NUTRIENT edges representing nutrient composition. This interconnected structure enables multi-hop traversal from diseases to nutrients and subsequently to candidate food items, forming the basis for graph-native retrieval and ranking during personalized nutrition recommendation.

Figure 4 illustrates the overall structure of the constructed knowledge graph. The graph integrates disease, nutrient, and food entities into a unified relational representation. Disease nodes are connected to relevant nutrient requirements, while food nodes encode nutrient composition information derived from the IFCT dataset. This multi-relational structure enables the system to perform multi-hop traversal from diseases to nutrients and subsequently to candidate food items, allowing the GraphRAG framework to compute personalized nutritional relevance directly within the graph.

### 5.2 Baseline models for comparison

To evaluate the effectiveness of the proposed GraphRAG framework, we compare its performance against five distinct baseline strategies representing the evolution of recommendation systems:

1. **Random recommendation (naive baseline):** A stochastic baseline that selects *k* food items uniformly at random from the database. It was implemented using a fixed random seed for reproducibility, drawing items from the IFCT 2017 without any nutritional filtering or ranking logic.
2. **Content-based filtering (CBF):** A traditional approach that recommends foods based on static nutrient attributes matching user thresholds. This was implemented using boolean filtering logic where food items are filtered based on absolute RDA caps for specific nutrients (e.g., Sugar < 5*g*) as defined by the user's demographic requirements as implemented in early systems like Evwiekpaefe et al. (2023), often failing to capture holistic dietary patterns.
3. **Collaborative filtering (CF):** A user-centric approach that predicts preferences based on historical similarity between users. In the absence of real interaction data, this was implemented using cosine similarity over synthetic user-food preference matrices derived from simulated nutrient alignment scores. As noted in the review by Bondevik et al. (2024), these models often struggle with the “cold-start” problem in health contexts where new users lack interaction history.
4. **Hybrid recommendation:** A fusion model combining content-based filtering with collaborative signals. Following the architecture by Chen et al. (2023), this was implemented by integrating the Boolean health constraints of CBF with the similarity scores of CF using a weighted average, applying health constraints as a post-retrieval mask.
5. **Standard LLMs (LLM Zero-Shot):** A direct prompting approach using models like GPT-4 or Gemini Pro without structured retrieval. Implementation involved a single-turn prompt containing only the user profile (age, gender, and stated condition) to generate meal suggestions without access to the structured IFCT or ICMR-NIN grounding datasets. As highlighted by Ase et al. (2025) and Parameswaran et al. (2025), while these models produce fluent text, they frequently “hallucinate” nutritional values or suggest culturally irrelevant items when lacking grounding data.

## 6 Behavioral and sensitivity analysis

### 6.1 Comparative behavioral analysis and statistical report

To quantitatively evaluate the effectiveness of the proposed GraphRAG framework, experiments were conducted across 20 diverse user profiles spanning variations in age, gender, and health conditions. For each profile, the system generated a ranked list of candidate food items based on disease-specific nutrient requirements derived from the knowledge graph.

Food items were considered nutritionally aligned if they satisfied at least two of the primary nutrient requirements associated with the target disease condition according to the knowledge graph constraints. Standard information retrieval metrics, including Precision@5 and NDCG@5, were computed using these relevance criteria. In addition, domain-specific metrics such as nutrient balance variance, demographic sensitivity (%DV deviation), ranking stability, and hallucination risk were evaluated to capture the clinical validity and robustness of the recommendations.

Table 11 summarizes the comparative performance of the proposed framework against baseline methods across these metrics. Higher NDCG and Precision indicate improved ranking quality, while lower balance variance and %DV deviation reflect better nutrient distribution and demographic alignment.

**Table 11:** Comprehensive statistical performance comparison (mean ± SD).

Method	NDCG@5	Precision@5	Balance variance	Demog. sens.	Ranking stability	Halluc. risk
Random recommendation	0.15 ± 0.04	0.10 ± 0.03	0.95	85.0	0.05	NA%
Content-based filtering	0.61 ± 0.05	0.55 ± 0.07	0.42 ± 0.08	45.3	0.65 ± 0.09	NA%
Collaborative filtering	0.58 ± 0.06	0.50 ± 0.08	0.78	62.1	0.72 ± 0.07	NA%
Hybrid recommendation	0.68 ± 0.04	0.62 ± 0.06	0.35 ± 0.05	38.5	0.81 ± 0.04	NA%
Standard LLMs	0.65 ± 0.07	0.60 ± 0.09	0.45	41.2	0.58 ± 0.12	14.2%
**Proposed GraphRAG**	**0.82** **±0.03**	**0.79** **±0.04**	**0.12** **±0.02**	**12.4**	**0.94** **±0.02**	**0.3%**

For Balance Variance, Demographic Sensitivity (%DV Deviation), and Hallucination Risk, lower values indicate better performance.

Higher NDCG, Precision values and Ranking Stability (RSS) indicate improved ranking quality.

This improvement can be attributed to graph-native constraint enforcement, RDA-based normalization, and deterministic ranking, which collectively promote balanced and nutritionally aligned recommendations.

- **Retrieval quality (NDCG and precision):** The results suggest that the GraphRAG framework indicates improved retrieval effectiveness within the evaluation setup compared to baselines. The higher NDCG indicates that the system supports the prioritization of the most nutritionally aligned food items at the top of the ranking.
- **Nutritional balance:** Constraint-aware and knowledge-based methods can explicitly encode dietary guidelines, giving them higher potential to optimize for balanced nutrient profiles. The significantly lower variance in the proposed method indicates a more proportional distribution of nutrients.
- **Demographic sensitivity:** Approaches that incorporate explicit rules or multi-objective health modeling appear to better account for age, sex, and clinical status specific RDA variations. The low deviation scores suggest that the GraphRAG recommendations are closely aligned with demographic-specific targets.
- **Ranking stability:** Deterministic, graph-structured models generally yield more reproducible rankings than purely interaction-based or free-form generative systems, as indicated by the high RSS values.
- **Hallucination risk:** LLM-only systems are prone to factual hallucinations in nutrition counseling, while retrieval-grounded approaches substantially reduce this risk by restricting the model to formatting validated graph data.

The results suggest that the GraphRAG framework indicates improved retrieval effectiveness within the evaluation setup compared to baseline methods. In particular, the higher NDCG@5 and Precision@5 values indicate that nutritionally aligned food items are more consistently ranked near the top of the recommendation list.

### 6.2 Performance latency analysis

We conducted an end-to-end latency test across 20 distinct query executions. The system architecture decouples the Retrieval Phase (Graph Traversal) from the Generation Phase (LLM Reasoning), resulting in a distinct bimodal latency profile.

As shown in Table 12, the core contribution of this paper (the Graph Retrieval Engine) is highly performant, retrieving and ranking foods in under 1 second. The observed delay (21*s* − 42*s* range) is strictly an artifact of the external Generative AI call. Future deployments can mitigate this by switching to smaller, locally hosted LLMs (e.g., Llama-3-8B) to bring total latency under 5 seconds.

**Table 12:** System latency breakdown (mean over 20 runs).

Component	Time (s)	Observation
Graph retrieval (local)	**< 1.2 s**	Real-time. Graph traversal and vector scoring are computationally efficient.
Cloud LLM generation (Gemini)	≈ 28.5 s	High latency due to external API overhead and token generation.
Total end-to-nd	≈ 29.7 s	Dominant factor is network/API constraint, not system architecture.

### 6.3 Quantitative stability and sensitivity analysis

To empirically validate the GraphRAG architecture, we focused on two critical metrics: Algorithm Sensitivity (Personalization) and Ranking Discrimination.

#### Algorithm sensitivity (proof of personalization)

We evaluated whether the system's mathematical core responds to demographic inputs by querying the same disease (Anemia) across three distinct user profiles: Male (15), Female (35), and Male (65).

We tracked the computed relevance scores for staple Indian foods like *Bengal Gram* and *Lentils* to observe vector shifts.

#### Analysis of personalization

As shown in Table 13, while the absolute rank of *Bengal Gram* remained stable at 50, the relevance score increased from 0.08 (Female 35) to 0.11 (Male 65). This 37% increase is numerically meaningful and reflects demographic sensitivity. It indicates that the Target Vector **V**_*target*_ in our cosine similarity formula is not static. It dynamically shifts based on the specific RDA requirements of the user (e.g., older males may have different protein-to-fiber ratios defined in the knowledge graph). A standard keyword search would yield identical scores (0.08) for all users; our system's varying scores confirm that personalization is mathematically active even for identical search queries.

**Table 13:** Sensitivity of relevance scores across demographics (anemia).

Food item	User profile	Rank	Relevance score	Interpretation
Bengal gram, whole	Female (35)	50	0.08	Baseline nutritional requirement for adult females
	Male (15)	50	0.09	Increased caloric and protein requirements during adolescence
	Male (65)	50	0.11	Adjusted micronutrient needs in older adults
Lentil, dal	Female (35)	47	0.09	Baseline nutritional requirement
	Male (15)	47	0.09	Comparable nutritional requirement across profiles
	Male (65)	47	0.12	Higher relevance due to age-specific micronutrient demands

#### Ranking discrimination (cosine distribution)

To verify that the recommendation engine effectively discriminates between high-quality and low-quality matches, we analyzed the score distribution of the Top-10 recommended foods for the Anemia condition.

The results shown in Table 14 demonstrate a sharp differentiation; the scaling does not affect relative ranking or comparative analysis. The top-ranked items achieve significantly higher scores (37.8) compared to the middle tier (24.0). This indicates the algorithm successfully isolates “Super-Matches” foods that offer superior nutrient density for the specific condition—rather than returning a flat list of generic items.

**Table 14:** Top-5 food score distribution (anemia).

Rank	Food item	Similarity score
1	Soya bean, white	37.81
2	Soya bean, brown	35.59
3	Fenugreek seeds	25.42
4	Eggs, cat fish	24.84
5	Tuna	24.58

Scores are scaled only for interpretability; all ranking logic operates on normalized cosine similarity. Similarity Scores have been scaled (×100) for easier interpretability.

**Ranking stability sensitivity analysis** To evaluate the robustness of the ranking engine, we conducted a sensitivity experiment where the target RDA values were perturbed by ±10%. This simulates minor variations in clinical guidelines or demographic attributes.

#### Analysis

The findings in Table 15 suggest that the system maintains high ranking stability even under minor shifts in nutritional targets. This indicates that the core retrieval logic remains robust against typical clinical or demographic variance, supporting the framework's reliability for decision support.

**Table 15:** Sensitivity analysis: impact of ±10% RDA perturbation on ranking.

Perturbation	Rank stability score (RSS)	Top-5 overlap
RDA +10%	0.96	100%
RDA –10%	0.94	90%

### 6.4 Nutrient coverage efficacy

Finally, we assessed the clinical utility of the recommendations. For an adult female with anemia (Target Iron: 29 mg/day), we aggregated the nutrient content of the top-10 recommended foods.

- **Total Iron Provided:** 47.04 mg
- **Daily Requirement Met: 162.2%**

The system not only meets but provides a safety margin over the daily requirement, ensuring that even with bioavailability losses (common in plant-based iron), the user receives a therapeutic dose.

### 6.5 Ablation study: effect of RDA capping and cosine similarity

This ablation experiment evaluates the specific computational impact of the RDA-capping mechanism and the vector-based ranking logic. By removing these components and reverting to a standard summation-based scoring method, we assess the framework's baseline behavior regarding “superfood bias”—a common failure mode where foods with extreme concentrations of a single nutrient dominate rankings despite poor overall balance.

#### Nutrient balance analysis

As shown in Table 16, the proposed GraphRAG framework exhibited significantly lower nutrient balance variance compared to the ablated summation-based method. The results suggest that the vector-based formulation, combined with deterministic capping, is a primary factor in promoting proportional nutrient distribution.

**Table 16:** Ablation study: impact of RDA capping on nutritional balance (mean ± SD).

Configuration	Balance variance	Observation
Without RDA capping (summation)	0.48 ± 0.09	High variance; “Superfood Bias” observed
With RDA capping (GraphRAG)	**0.12** **±0.02**	Low variance; proportional balance

Bold values denote better performance (lower balance variance).

#### Nutrient coverage and bias mitigation

The findings indicate that foods ranked highly by the proposed framework satisfy a broader set of disease-specific nutrient requirements (higher Nutrient Coverage Score) compared to the baseline. Without the capping mechanism, nutrient-dense items such as Liver or Agathi leaves tend to dominate the top rankings due to single-nutrient spikes, even when they fail to meet a user's broader clinical profile. The results suggest that the proposed ranking strategy helps reduce this dominance, supporting the generation of more balanced and nutritionally appropriate recommendations. To contextualize these performance indicators: higher NDCG and Precision values indicate improved ranking quality and relevance, while lower nutrient balance variance reflects better, more proportional nutrient distribution across the recommended meal. Additionally, a lower %DV deviation indicates stronger demographic alignment with the user's specific RDA targets.

### 6.6 Qualitative comparison with LLM-only systems

LLM-only approaches were evaluated qualitatively due to their lack of structured nutritional constraints. Observed limitations included:

- Generic dietary advice lacking demographic specificity,
- Inconsistent prioritization of nutritionally aligned nutrients, and
- Elevated risk of hallucinated or unsafe recommendations.

By contrast, the proposed GraphRAG system consistently produced fact-grounded, explainable, and demographically sensitive recommendations, with generative models strictly constrained to post-retrieval formatting.

The only existing work in meal planning that combines a knowledge graph and an LLM is FKG.in by Gupta et al. (2024a); a detailed comparison between the proposed GraphRAG and FKG.in is given in Table 17.

**Table 17:** Comparative analysis: proposed GraphRAG framework vs. FKG.in enhancement framework.

Dimension	FKG.in enhancement (Gupta et al., 2024a)	Proposed GraphRAG framework
Primary objective	Knowledge curation Focuses on extracting and estimating nutrient data from unstructured recipes to build a digital food library.	Clinical intervention Focuses on generating personalized, nutritionally safe meal plans by satisfying strict disease-specific constraints.
Clinical grounding	Nutritional analysis Aggregates composition data (IFCT, INDB). Focuses on food chemistry rather than disease pathology.	Pathology-aware Explicitly models 70+ diseases, linking specific symptoms to nutrient requirements via medical guidelines.
Thali/Indian datasets	Recipe-centric Aggregates 25,000+ recipes from blogs. Solves the challenge of unstructured text and ingredient variations.	Structure-centric Models the “Indian Thali” meal structure. Uses strict RDA standards (ICMR-NIN Expert Committee, 2020) for personalization.
Knowledge graph usage	Data repository Acts as a storage system for recipes, ingredients, and computed nutrient values.	Reasoning engine Acts as the logical core. Executes vector similarity searches directly within the graph to rank foods.
RAG/ Generative AI	LLM as extractor Uses LLMs (GPT-3.5) to parse text, translate vernacular terms, and estimate ingredient weights.	LLM as formatter Uses LLMs (Gemini) *strictly* to format graph outputs into natural language. Prohibits nutritional reasoning.
Hallucination control	Human-in-the-loop Acknowledges risks in LLM estimation; relies on manual verification during data ingestion.	Deterministic retrieval Mitigates hallucination by separating reasoning (graph) from generation (LLM). Values are retrieved, not guessed.
Explainability	Data provenance Tracks the source of the data (e.g., “Sourced from LLM” vs. “Sourced from IFCT”).	Reasoning traceability Explains *why* a recommendation was made by tracing the Disease → Nutrient → Food path.

### 6.7 Summary of findings

The experimental and comparative evaluation demonstrates that the proposed GraphRAG framework:

- Produces nutritionally balanced and stable rankings,
- Responds meaningfully to demographic-specific dietary requirements,
- Eliminates superfood bias through vector-based ranking, and
- Maintains safety and explainability by limiting LLM usage to formatting.

These findings validate the effectiveness of graph-native computation for personalized, culturally grounded nutrition recommendation.

## 7 Discussions and implications of the study

The experimental findings provide evidence supporting the research questions outlined in Section 1. First, the results demonstrate that disease-specific nutritional requirements can be effectively linked to culturally grounded meal structures such as the Indian Thali through a unified knowledge graph representation. By modeling explicit relationships among diseases, nutrients, and food ingredients, the framework translates clinical dietary constraints into interpretable meal recommendations. Second, the quantitative evaluation indicates that graph-native ranking enables more stable and nutritionally balanced recommendations than traditional filtering or LLM-only approaches, as reflected by improved ranking metrics and lower nutrient variance. Finally, the constrained Graph–RAG architecture illustrates how generative models can be safely integrated into nutrition recommendation pipelines when the reasoning process remains grounded in structured knowledge. Together, these observations suggest that knowledge-graph–driven retrieval combined with controlled language generation can support transparent and culturally relevant personalized nutrition planning. The framework is intended as a decision-support tool and not as a substitute for professional medical or dietary advice.

We now discuss the experimental findings and examine their potential technical, clinical, and societal implications.

### 7.1 GraphRAG performance factors

The proposed framework indicates promise in improving over existing approaches due to its graph-based design, which appears to enhance ranking stability, demographic sensitivity, and nutritional balance. Rather than treating nutrients as isolated features, this approach models nutrition as an interconnected problem encompassing diseases, nutrients, foods, and demographic factors. By executing multi-hop reasoning directly within the Neo4j knowledge graph, the system supports the maintenance of contextual integrity throughout the recommendation process. This contrasts with flat-table approaches, which can struggle to preserve relational dependencies. The results suggest that graph-native computation is well-suited for domains where correctness depends on the interaction of multiple interdependent factors.

### 7.2 Addressing superfood bias through vector-based balance

A key insight from the analysis is the importance of explicitly modeling nutritional balance. Summation-based ranking approaches often favor foods with extreme nutrient concentrations, which can result in repetitive recommendations. The cosine similarity–based ranking formulation helps address this limitation by comparing each food's nutrient contribution vector against an idealized profile. By capping excessive nutrient contributions and prioritizing vector alignment, the system supports the selection of foods that collectively satisfy dietary needs. This formulation aligns with nutritional science principles and indicates a potential reduction in superfood bias.

### 7.3 Hallucination risk and safety

Comparing with LLM-only systems highlights a safety issue in AI nutrition advice: hallucinations that cannot be verified. The proposed system aims to mitigate this risk by design. All nutritional reasoning is performed deterministically within the knowledge graph, which serves as a single source of truth. The generative model is restricted to a post-retrieval formatting role, which supports the grounding of all outputs in validated data. This separation of reasoning and generation appears particularly important in healthcare contexts where accountability is essential.

### 7.4 Cultural relevance and user adherence

The results suggest that cultural context is important for dietary adherence. By using Indian foods and the traditional Thali structure, the system helps avoid the cultural mismatch often seen in Western-focused apps. Recommendations that feel familiar may be more useful to users, indicating that cultural fit is a key part of making personalized nutrition effective for diverse groups.

### 7.5 Broader implications

The results of this study support the potential of the GraphRAG framework as a generalizable blueprint for safety-critical domains. By anchoring reasoning in structured knowledge, similar architectures could be applied to nutritional decision support or drug–nutrient interaction analysis. This transparency indicates a pathway for clinicians to validate and adjust suggestions, making the tool a practical option for real-world decision support rather than automated prescribing.

### 7.6 Implications for hybrid AI systems

Beyond the specific application to personalized nutrition, the proposed framework illustrates a general design pattern for hybrid AI systems that combine structured reasoning with controlled generative models. By anchoring the decision logic within a knowledge graph and restricting the language model to post-retrieval explanation and formatting, the architecture separates reasoning from generation. This separation improves interpretability, reduces hallucination risk, and allows domain constraints to be enforced directly within the graph structure. Such an approach can be extended to other recommendation and decision-support domains where domain knowledge, safety constraints, and explainability are essential, including healthcare analytics, clinical decision support, and policy-aware recommendation systems.

While these findings highlight the potential of graph-driven reasoning for personalized nutrition recommendation, several limitations of the current study should also be acknowledged.

## 8 Limitations and future work

The proposed GraphRAG framework demonstrates promising results; however, several limitations must be acknowledged.

First, the evaluation is conducted within a controlled, graph-defined environment using constructed user profiles and relevance criteria. While this enables systematic and reproducible analysis of system behavior, it limits external validity. The study does not incorporate real-world user interaction data, clinical expert validation, or standardized benchmark datasets. Consequently, the reported results primarily reflect internal consistency, ranking stability, and adherence to encoded nutritional constraints rather than validated clinical effectiveness. The deterministic formulation does not explicitly model uncertainty or interdependencies between nutrients, which may influence real-world dietary outcomes.

Second, the knowledge graph is constructed primarily from IFCT 2017 and associated datasets. Although these sources provide reliable and culturally grounded nutritional information, the coverage remains limited to the included food items and nutrient mappings. Regional variations, evolving dietary practices, and emerging food alternatives may not be fully represented. Expanding the knowledge base with additional datasets and region-specific food repositories would improve coverage and applicability.

Third, the evaluation is performed on a limited set of user profiles and selected chronic conditions. While this supports analysis of system behavior and demographic sensitivity, larger-scale validation across diverse populations is required to assess generalizability. Future work should include broader simulations, user studies, and real-world deployment scenarios.

Fourth, the system is designed as a decision-support tool rather than a fully autonomous clinical system. Although recommendations are grounded in nutritional guidelines, they should be interpreted as supportive suggestions and used in conjunction with professional medical advice. The absence of clinical trials or longitudinal validation studies limits the ability to assess real-world health outcomes and user adherence.

Finally, the current implementation relies on an external cloud-based language model for natural language generation. While the model is restricted to formatting validated outputs, this introduces latency overhead and dependency on external services. Future implementations may explore lightweight or locally deployed models to improve efficiency and support real-time applications.

Addressing these limitations through expanded datasets, real-world validation, and improved system deployment will be critical for advancing graph-based AI systems in personalized nutrition.

### 8.1 Deterministic design and uncertainty considerations

The proposed framework adopts a deterministic, graph-based ranking approach that prioritizes interpretability, reproducibility, and controlled reasoning. By constraining all nutritional inference within a structured knowledge graph, the system ensures that each recommendation can be directly traced to explicit disease–nutrient–food relationships and demographic-specific RDA constraints. This design choice reduces ambiguity in the decision process and avoids the stochastic variability commonly associated with purely generative or data-driven models, thereby supporting transparency and consistency in recommendation outcomes. The deterministic design was intentionally adopted to prioritize transparency, reproducibility, and traceability over probabilistic flexibility.

However, this deterministic formulation introduces important limitations. Nutritional requirements and dietary responses are inherently context-dependent and influenced by individual variability, comorbidities, lifestyle factors, and incomplete or uncertain knowledge. The current framework does not explicitly model such uncertainty, nor does it provide confidence estimates or probabilistic interpretations of recommendations. As a result, the system may not fully capture variability in real-world scenarios, and its outputs should be interpreted as structured, guideline-aligned suggestions rather than definitive or clinically validated prescriptions.

Future work could address these limitations by incorporating uncertainty-aware mechanisms into the framework. Potential directions include the integration of probabilistic knowledge graphs, Bayesian inference models, or hybrid approaches that combine deterministic reasoning with probabilistic scoring to represent confidence and variability. Such extensions would enable the system to better reflect the inherent uncertainty in nutritional science while preserving the interpretability advantages of graph-based reasoning.

This limitation is particularly significant in clinical contexts where uncertainty and patient variability directly influence dietary outcomes.

### 8.2 Ethical and practical considerations

The deployment of AI-driven nutrition recommendation systems introduces several important ethical and practical considerations. First, the proposed framework is designed as a decision-support tool and not as a clinical diagnostic or prescriptive system. The system is not intended to replace professional medical or dietary advice. Over-reliance on automated recommendations without appropriate professional oversight may lead to suboptimal or potentially inappropriate dietary decisions, particularly for individuals with complex medical conditions, multiple comorbidities, or acute health concerns.

Second, the underlying knowledge graph is constructed from curated datasets that may inherently reflect cultural, regional, and dietary biases. While the system is tailored toward the Indian context, this specialization may limit its generalizability across diverse populations with differing dietary practices, nutritional requirements, or food availability. Additionally, the framework assumes accurate and complete user input; however, real-world usage may involve incomplete, imprecise, or ambiguous information, which can affect the quality and relevance of recommendations.

Furthermore, the system does not explicitly account for dynamic factors such as lifestyle variations, metabolic differences, or evolving clinical conditions, which are critical in real-world dietary planning. As a result, the generated recommendations should be interpreted as structured, guideline-aligned suggestions rather than definitive or clinically validated prescriptions.

In addition, the deployment of AI-driven nutrition recommendation systems raises several important ethical and practical considerations. First, there is a risk of over-reliance on automated recommendations, particularly when users interpret system outputs as authoritative guidance rather than decision-support suggestions. This may lead to inappropriate dietary choices, especially for individuals with complex medical conditions that require personalized clinical oversight. Second, the knowledge graph and underlying datasets may embed implicit biases, including cultural, regional, and dietary assumptions derived from the data sources (e.g., Indian food composition tables and standardized dietary guidelines). Such biases may influence the recommendations and limit their neutrality across different dietary practices. Third, the applicability of the system across diverse populations is inherently constrained, as nutritional requirements, food availability, cultural preferences, and health conditions vary significantly across regions and demographic groups. Consequently, recommendations generated within one cultural or nutritional context may not generalize effectively to others. Therefore, the proposed framework should be interpreted as a structured, knowledge-guided decision-support tool rather than a universally applicable solution, and its outputs should be used in conjunction with expert judgment and contextual considerations.

Given these considerations, the framework is best suited for use in supportive contexts, such as preliminary dietary guidance or educational purposes, and should be applied in conjunction with domain expertise from healthcare professionals. Future work should explore mechanisms for bias mitigation, user-in-the-loop validation, and integration with clinical workflows to ensure safe and responsible deployment.

## 9 Conclusions

This work indicates the potential of GraphRAG architectures for constraint-driven recommendation tasks by introducing a framework for creating personalized and culturally relevant nutrition planning. The approach integrates a Neo4j knowledge graph with a deterministic retrieval pipeline to support the generation of demographically tailored dietary advice.

The primary contribution of this work lies in the architectural integration of graph-native reasoning with constrained language generation, rather than in establishing clinically validated outcomes. It aids in modeling nutrition as a structured, relational problem, which helps avoid the inconsistencies often associated with unconstrained generative or purely feature-based methods. By mapping explicit links between diseases, nutrients, foods, and age- and gender-specific RDAs, the system supports reliable reasoning and helps reduce common issues observed in heuristic models.

Evaluation across 20 diverse user profiles suggests that the framework maintains high ranking stability and clinical alignment. Quantitative results indicate an NDCG@5 of 0.82 ± 0.03, suggesting an improvement in retrieval effectiveness over baseline content-based and hybrid models. Furthermore, results from the ablation study indicate that the inclusion of RDA capping and cosine similarity helps reduce superfood bias, supporting a more proportional distribution of nutrients. By restricting the language model to a post-retrieval formatting role, the architecture effectively reduces the risk of hallucination while supporting the explainability of the recommendation pathway.

Beyond the immediate application to Indian dietary planning, this work highlights the broader potential of GraphRAG architectures in healthcare and other safety-critical domains. The separation of structured reasoning from NLG provides a practical pathway toward trustworthy and explainable AI systems that can support, rather than replace, human decision-making.

Future work will focus on addressing current methodological limitations by conducting formal clinical trials and expanding the knowledge graph to include seasonal variations and longitudinal meal planning. Additionally, evaluating the system across the full spectrum of the 70 modeled disease conditions and integrating real-time user feedback will further support the development of fair, reliable, and culturally adaptive precision nutrition tools. Together, these steps aim to make AI-driven nutrition more equitable and clinically grounded.

## Data Availability

The original contributions presented in the study are included in the article/Supplementary material, further inquiries can be directed to the corresponding author.
