# Supplementary material for: An explainable graph retrieval augmented generation framework for personalized nutrition recommendation
Source: Front Artif Intell. 2026 Apr 30;9:1808444. doi: 10.3389/frai.2026.1808444 (PMC13171842; doi:10.3389/frai.2026.1808444)
Supplement: Supplementary file 1 [file Supplementary_file_1.pdf]

frontiers

Supplementary Material

IMPLEMENTATION DETAILS AND REPRODUCIBILITY GUIDE

Environment Configuration: The system initializes its execution environment through secure configuration of external dependencies. API credentials (e.g., Google Gemini API keys) are managed via environment variables to ensure security and portability. The backend includes validation checks and exception-handling mechanisms to gracefully handle missing or invalid configurations, thereby preventing runtime failures.

Data Pipeline and System Dependencies: The Smart ETL pipeline is implemented through a custom script, `migrate_to_graph.py`, which orchestrates data ingestion, normalization, and graph construction. The following core libraries are used:

- **Pandas:** Handles structured data processing, including CSV parsing, cleaning, and normalization of nutrient values.
- **SentenceTransformers:** The `all-MiniLM-L6-v2` model is used to generate 384-dimensional embeddings for semantic alignment between clinical and nutritional terminology.
- **Neo4j:** Serves as the graph database for storing and querying disease–nutrient–food relationships using Cypher queries.
- **FastAPI:** Provides an asynchronous backend framework for handling user queries and integrating the GraphRAG pipeline.
- **Torch:** Supports embedding generation and model inference operations.

Reproducibility Workflow: To facilitate reproducibility, the system can be deployed using the following step-by-step procedure:

1. **Install Dependencies:** Install Python (3.9+) and required libraries using `pip install -r requirements.txt`. Ensure Neo4j Community Edition is installed and running locally.
2. **Database Setup:** Initialize a Neo4j instance and configure connection credentials (URI, username, password) via environment variables.
3. **Dataset Preparation:** Download and organize the required datasets (IFCT 2017, ICMR-NIN 2020, and disease–nutrient mappings) into the designated data directory.
4. **Graph Construction:** Execute the ETL pipeline using:
   ```
   python migrate_to_graph.py
   ```
   This step performs semantic normalization, entity mapping, and populates the Neo4j knowledge graph.
5. **API Deployment:** Launch the FastAPI server:
   ```
   uvicorn main:app --reload
   ```
   This enables real-time interaction with the GraphRAG system.
6. **Query Execution:** Submit user queries via API endpoints or a client interface. The system processes input through semantic mapping, graph retrieval, and ranking.

7. **Evaluation Pipeline:** Run evaluation scripts to reproduce reported metrics (Precision@k, NDCG@k, RSS, etc.) using predefined user profiles and scenarios.

Implementation Notes: The system is platform-independent and can be executed on standard development machines. While GPU acceleration is optional, it is recommended for faster embedding computations. The modular design of the ETL pipeline and ranking engine allows independent testing and extension of each component.

Code availability: The source code and implementation details are available at `https://github.com/DecoderOP/Indian-thali`.
